# Transcriptomic Insights into Benzenamine Effects on the Development, Aflatoxin Biosynthesis, and Virulence of *Aspergillus flavus*

**DOI:** 10.3390/toxins11020070

**Published:** 2019-01-27

**Authors:** Mingguan Yang, Laifeng Lu, Shuhua Li, Jing Zhang, Zhenjing Li, Shufen Wu, Qingbin Guo, Huanhuan Liu, Changlu Wang

**Affiliations:** State Key Laboratory of Food Nutrition and Safety, Key Laboratory of Food Nutrition and Safety, Ministry of Education, College of Food Engineering and Biotechnology, Tianjin University of Science and Technology, Tianjin 300457, China; minghuang_2013@163.com (M.Y.); Frank@tust.edu.cn (L.L.); lishuhua05@163.com (S.L.); 18733520963@163.com (J.Z.); lizhenjing@tust.edu.cn (Z.L.); wushufen@tust.edu.cn (S.W.); guoqingbin008322@tust.edu.cn (Q.G.); lh_tust@tust.edu.cn (H.L.)

**Keywords:** *Aspergillus flavus*, aflatoxin B1, benzenamine, fumigation, transcriptome

## Abstract

*Aspergillus flavus* is a soilborne pathogenic fungus that poses a serious public health threat due to it contamination of food with carcinogenic aflatoxins. Our previous studies have demonstrated that benzenamine displayed strong inhibitory effects on the mycelial growth of *A. flavus*. In this study, we systematically investigated the inhibitory effects of benzenamine on the development, aflatoxin biosynthesis, and virulence in *A. flavus*, as well as the underlying mechanism. The results indicated that benzenamine exhibited great capacity to combat *A. flavus* at a concentration of 100 µL/L, leading to significantly decreased aflatoxin accumulation and colonization capacity in maize. The transcriptional profile revealed that 3589 genes show altered mRNA levels in the *A. flavus* after treatment with benzenamine, including 1890 down-regulated and 1699 up-regulated genes. Most of the differentially expressed genes participated in the biosynthesis and metabolism of amino acid, purine metabolism, and protein processing in endoplasmic reticulum. Additionally, the results brought us to a suggestion that benzenamine affects the development, aflatoxin biosynthesis, and pathogenicity of *A. flavus* via down-regulating related genes by depressing the expression of the global regulatory factor *leaA*. Overall, this study indicates that benzenamine have tremendous potential to act as a fumigant against pathogenic *A. flavus*. Furthermore, this work offers valuable information regarding the underlying antifungal mechanism of benzenamine against *A. flavus* at the level of transcription, and these potential targets may be conducive in developing new strategies for preventing aflatoxin contamination.

## 1. Introduction

*Aspergillus flavus*, an opportunistic pathogen of both humans and plants, produces an abundance of diverse secondary metabolites, including aflatoxins. Aflatoxins are the most important mycotoxin due to their common occurrence among the serious threats that are posed to humans and animals. About 18 different types of aflatoxin are now known [1]. Among these, aflatoxin B1 is regarded as the most potent natural carcinogen and it is classified as a Group I carcinogen by the International Agency for Research on Cancer (IARC) [2]. It is estimated that up to 28% of all hepatocellular carcinoma cases worldwide may be caused by aflatoxins [3].

*A. flavus* is the primary etiological agent of aflatoxin contamination of agricultural commodities, such as corn and peanut [4]. The Food and Agriculture Organization (FAO) forecasts that approximately 2595 million tonnes of cereals will be produced and 2649 million tonnes will be consumed in 2018 [5]. In addition, cereal losses due to other factors, including climate-related natural disasters and conflict, have increased the prevalence of undernourishment. The estimated number of undernourished people increased to nearly 821 million in 2017 [6]. Therefore, preventing aflatoxin contamination is necessary in addressing the problem of food shortage and food safety.

To minimize the harmful effects of aflatoxins, several strategies have been developed to control toxigenic fungus growth and aflatoxin production. Volatiles, such as aldehyde, acetate esters, and alcohols of plant and microbial origin, have been shown to strongly inhibit toxigenic fungus growth and aflatoxin formation [7,8,9,10]. Fumigation with natural volatiles is an ideal method in controlling *A. flavus*, as it ensures that food is protected from pathogenic fungi with reduced or no organoleptic changes [11]. Furthermore, volatiles are easily volatilized at ambient temperature. This characteristic gives volatile compounds a great advantage from the point of view of application practicality and homogeneity [12]. Among these volatiles, ethers, such as dimethyl disulfide and dimethyl sulfide, have been proved to be effective agents for combating pathogens [13,14]. Previously, we demonstrated that benzenamine has great capacity for controlling the growth of *A. flavus* [15]. However, the inhibitory effects have not yet been studied in depth. It is not clear whether aflatoxin production is affected, and the underlying mechanisms are not known.

The genomes of several species of *Aspergillus* have recently been sequenced and analyzed, and the regulation of aflatoxin biosynthesis and development in *A. flavus* has been well studied [16]. The biosynthetic pathway of aflatoxins has been essentially clarified [17]. In addition, the functions of several global regulatory genes, such as *laeA* and *veA*, which are involved in fungal secondary metabolism and development, have been characterized [18,19]. High-throughput sequencing technologies are currently revolutionizing the field of biology and RNA sequencing (RNA-Seq) has been applied to study a range of eukaryotic transcriptomes, with less sampling bias, higher resolution, and much broader expression range coverage [20,21].

In this study, we are interested in revealing the antimicrobial activity of benzenamine against *A. flavus*. The RNA-Seq approach was applied to systematically investigate the mechanism of benzenamine-induced regulation of the development, aflatoxin biosynthesis, and virulence of *A. flavus*. This work will be meaningful for further understanding the interactions of volatiles with *A. flavus* and the regulation of aflatoxin biosynthesis, and the results should be of interest to those that are studying the management of *A. flavus* contamination in agricultural products.

## 2. Results and Discussion

### 2.1. Antagonistic Activity of Benzenamine against A. flavus

**Figure 1:**
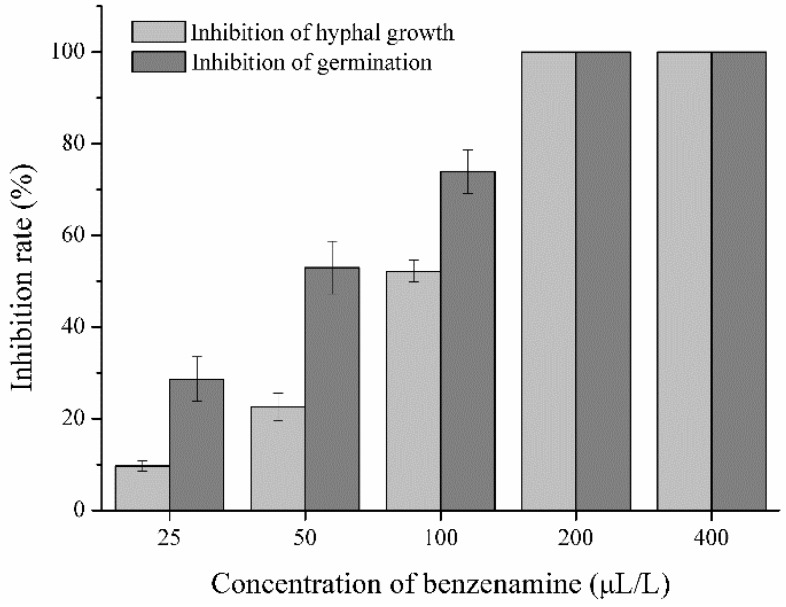
Effects of benzenamine on the hyphal growth and spore germination of *Aspergillus flavus*. The applied concentrations of benzenamine were 25, 50, 100, 200, and 400 µL/L. Results are presented as the mean ± SD.

Fungal colony diameter, aflatoxin production, and colonization of maize were quantified to define the inhibitory effect of benzenamine in the development, toxigenicity, and virulence of *A. flavus*. As shown in Figure 1, benzenamine exerted inhibitory effects on the mycelial growth and spore germination of *A. flavus* at the tested concentrations. Increasing concentrations of benzenamine (from 25 to 400 μL/L) resulted in a significant increase in growth inhibition (from 9.67 to 100%). Untreated mycelia grew to a diameter of 4.60 cm by three days post-inoculation, and conidia germinated completely within 9 h. The inhibition of hyphal growth and conidial germination of *A. flavus* resulting from treatment with 100 µL/L of benzenamine was 52.19% and 73.96%, respectively. Additionally, the minimum inhibitory concentration (MIC) of benzenamine against *A. flavus* was found to be 200 μL/L. The growth and conidial germination of *A. flavus* were completely inhibited at this concentration. Interestingly, we noted that exposing *A. flavus* to benzenamine for three days inhibited the fungus, but it renewed its growth after being transferred into fresh Potato Dextrose Agar (PDA) plates. This phenomenon clearly indicates that benzenamine suppressed *A. flavus* growth but did not kill *A. flavus*.

**Figure 2:**
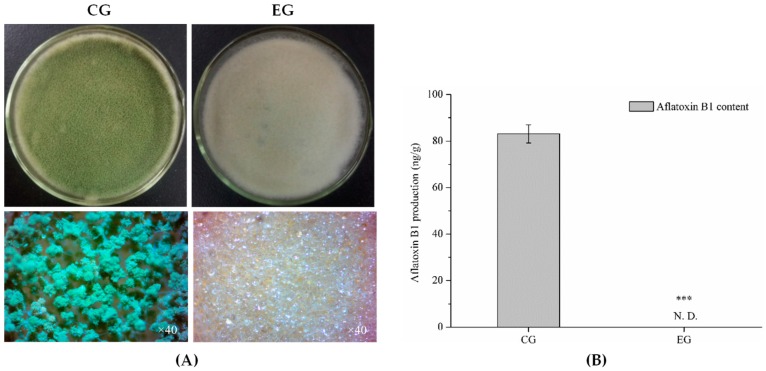
Effects of 100 µL/L of benzenamine on aflatoxin production. (**A**) Morphological characterization of *Aspergillus flavus* in the absence (CG) and presence (EG) of benzenamine. (**B**) Aflatoxin B1 accumulation by *Aspergillus flavus* in the absence (CG) and presence (EG) of benzenamine. The results are presented as mean ± SD. Asterisks indicate a significant difference between groups (*** *p* < 0.001), N. D. denotes not detected (<0.03 ng/g).

**Figure 3:**
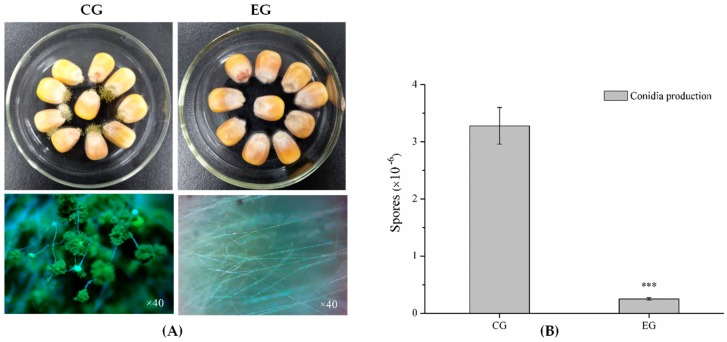
Effects of 100 µL/L of benzenamine on *Aspergillus flavus* infection in maize. (**A**) CG: maize inoculated with *Aspergillus flavus* at five days post-inoculation; EG: maize inoculated with *Aspergillus flavus* exposed to benzenamine for five days. (**B**) The production of *Aspergillus flavus* conidia on maize in CG and EG. Results are presented as the mean ± SD. Asterisks indicate a significant difference between groups (*** *p* < 0.001).

Subsequently, 100 μL/L of benzenamine with moderate bioactivity was applied to further investigate the inhibitory effect of benzenamine on the toxigenicity and virulence of *A. flavus*. Figure 2 shows the effect of benzamine treatment on aflatoxin B1 production. The concentration of aflatoxin B1 was 83.14 ng/g in control groups (CG), whereas no aflatoxin B1 (<0.03 ng/g) was detected in *A. flavus* that was treated with benzenamine (EG—experimental group). The results for maize that was colonized by *A. flavus* are shown in Figure 3. In untreated maize kernels (CG), inoculation with *A. flavus* caused the complete colonization (3.28 × 10^6^ conidia/mL) within five days. In the treatments exposing infected kernels to 100 μL/L of benzenamine (EG), no visible symptoms were observed, and the number of conidia sharply decreased to 0.25 × 10^6^ conidia/mL. The results of the antifungal ability experiment clearly indicate that benzenamine displays strong inhibitory effects on the development, aflatoxin biosynthesis, and fungal virulence of *A. flavus*.

### 2.2. Transcriptome Overview

To identify *A. flavus* genes that were differentially regulated during continuous exposure to benzenamine, a transcriptome analysis of *A. flavus* with three biological replicates was performed using the Illumina platform. Raw sequencing data can have issues regarding low quality, which can significantly distort analytical results and lead to erroneous conclusions. Therefore, quality control steps were performed to ensure that RNA-Seq data were of high quality. The clean reads were obtained by trimming the raw data containing adapters, poor-quality bases (<Q20), and a sequence length smaller than 50 nucleotides (Table S1). After the assembly of clean data, a total of 23,639 unigenes were obtained, with a mean length of 1437 bp (Table S2).

### 2.3. Annotation and Analysis of All Unigenes

To understand the transcriptome of *A. flavus*, all of the unigenes were aligned against several databases using BLASTx (*E*-value ≤ 10^–5^), including NR (NCBI non-redundant protein sequences), GO (Gene Ontology), KEGG (Kyoto Encyclopedia of Genes and Genome), eggNOG (evolutionary genealogy of genes: Non-supervised Orthologous Groups), and Swiss-Prot. The results are summarized in Table S3. A total of 14,684 unigenes were matched to known proteins in the NR database, although the genome of *A. flavus* is estimated to contain 13,485 genes [22]. This may be due to the variety in the transcripts from processes, such as alternative splicing and posttranscriptional regulation [23]. The results indicate that more unigenes have the potential for translation into functional proteins, which serves to improve the annotation of the *A. flavus* genome.

### 2.4. Functional and Pathway Enrichment Analysis of Differentially Expressed Genes

**Figure 4:**
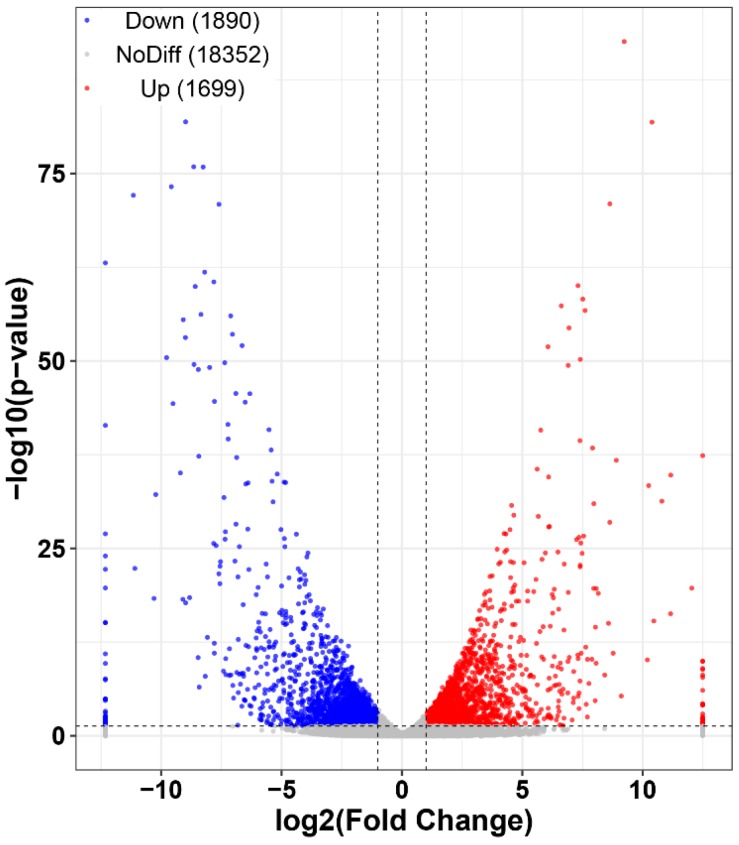
Number of genes showed up-regulated and down-regulated expression in experimental group (EG) vs. control group (CG).

**Figure 5:**
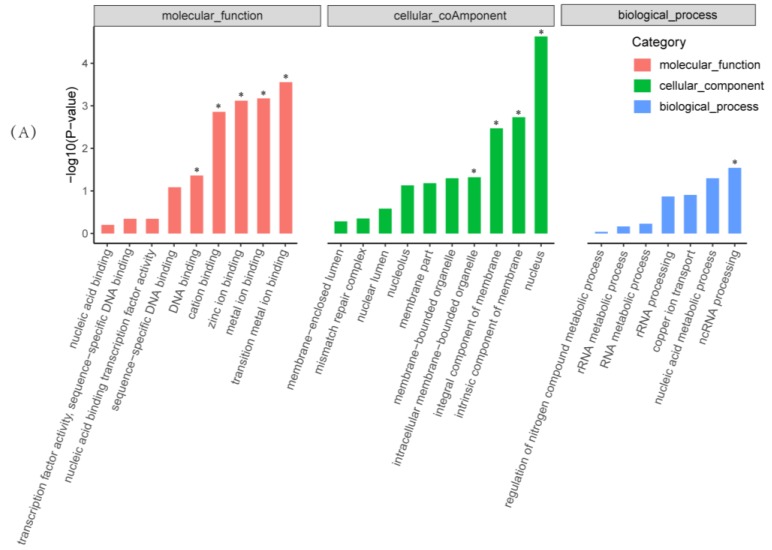

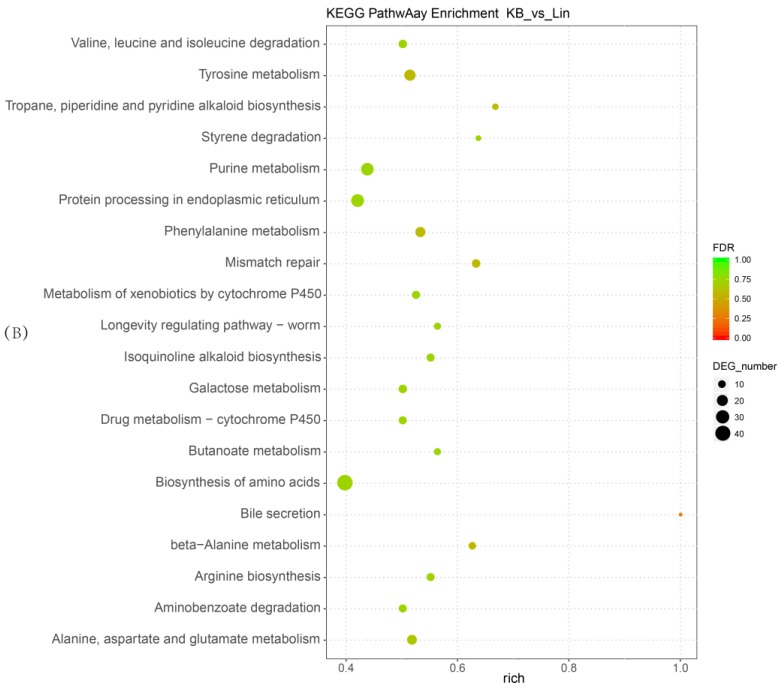
Gene Ontology (GO) functional classification and Kyoto Encyclopedia of Genes and Genome (KEGG) pathway enrichment of differentially expressed genes when *Aspergillus flavus* was treated with benzenamine. (**A**) Functional categories at three developmental stages based on GO enrichment analysis of differentially expressed genes (DEGs), and the asterisk means significant enrichment (* corrected *p*-value <0.05). (**B**) KEGG pathway enrichment analysis of DEGs.

Out of the 23,639 unigenes, 3589 showed differential accumulation of mRNAs. Among all differentially expressed genes (DEGs), 1890 genes (accounting for 52.66% of all DEGs) were significantly downregulated and 1699 genes (accounting for 47.34% of all DEGs) were upregulated compared with the untreated samples (Figure 4, Table S4). All DEGs were subjected to GO and KEGG enrichment analyses. A total of 2539 DEGs were mapped to 2836 GO terms. Among these, 1773, 653, and 410 GO terms belong to the biological process, molecular function, and cellular component categories, respectively. As shown in Figure 5A, transition metal ion binding, metal ion binding, zinc ion binding, cation binding, and DNA binding are significant enrichment terms that belong to the molecular function category. The significant functional terms in the cellular component category are related to the nucleus, the intrinsic/integral component of the membrane, and intracellular membrane-bounded organelles. Additionally, ncRNA processing and the nucleic acid metabolic process are the most abundant in the biological process category. According to the KEGG pathway database, significantly enriched pathways include the biosynthesis and metabolism of amino acids (tyrosine metabolism; phenylalanine metabolism; alanine, aspartate, and glutamate metabolism; etc.), purine metabolism, protein processing in the endoplasmic reticulum, and mismatch repair, amongst others (Figure 5B). Thus, the RNA-Seq results indicate that benzenamine exerts complex regulatory effects on *A. flavus*. Next, the genes that are involved in the development, aflatoxin biosynthesis, and virulence of *A. flavus* were further analyzed.

### 2.5. Analysis of DEGs Involved in Development

To elucidate the effects of benzenamine on the development of *A. flavus*, DEGs that are related to the cell wall, cell membrane, conidia, transcription factors, and others were analyzed in our work (Table 1).

#### 2.5.1. DEGs Involved in the Cell Wall

The cell wall provides fungi with a protective barrier against environmental stresses, and it is essential for the survival of the fungus during development and reproduction [24]. It has been reported that the cell wall is an important molecular target of antifungal compounds [25]. Numerous DEGs that are involved in the cell wall were found in this study (Table 1).

α-1,3-Glucan and β-1,3-Glucan play critical roles in maintaining the normal morphology of the fungal cell wall. *Ags1* encodes a synthase that mediates the synthesis of α-1,3-glucan [26]. The enzyme β-1,3-glucan synthase, encoded by *fks1*, is an essential and unique structural component of β-1,3-Glucan [27]. In the current work, *ags1* and *fks1* were significantly downregulated by benzenamine. Chitin is an important structural polysaccharide of the cell wall, and chitin synthesis is directly governed by chitin synthase [28]. The transcription of chitin synthase genes *chs6* and *chs8* was moderately downregulated. Chitinase is conducive to fungal cell separation during their reproduction period. The downregulation of the glucanase gene *crh11* was also found, and this could lead to fungal reproduction disorder [29].

Glycosylphosphatidyl-inositol (GPI)-anchored proteins are one of the major cell wall components. GPI-anchored proteins are essential for the normal function of glucan assembly [30]. The genes that are responsible for GPI-anchored protein biosynthesis are potential targets of antifungal reagents. The RNA-Seq data show that *cfmA* (GPI-anchored CFEM domain protein) and *afuA* (GPI-anchored membrane protein) had reduced expression in our study. In addition, the regulatory subunit of the *rho* family of GTPases is essential to the cell wall integrity signaling pathway. It has been proved that the deletion of the *rho* protein results in cytoplasmic leakage [25]. Interestingly, the GTP-binding proteins *rho2* and *rho4* had 2.68- and 2.48-fold increases in expression, respectively. This phenomenon may be a defensive response of cells to overcome external stimulation [31].

#### 2.5.2. DEGs Involved in the Cell Membrane

The cell membrane plays important roles in the maintenance of osmotic pressure and normal physiological function; thus, it is another important target of the antimicrobial substance [32]. Ergosterol is an important and specific component of the fungal cell membrane, and it is essential for fungal growth and development [33]. Ergosterol is considered to be crucial in regulating cell membrane fluidity, permeability, and membrane-bound enzyme activities, as well as in substance transportation [34]. Furthermore, ergosterol can stimulate the growth and proliferation of fungi [35]. As shown in Table 1, eight ergosterol biosynthesis genes displayed significant expression. Of these eight, seven DEGs were downregulated, exhibiting fold changes that ranged between 2.35- and 82.03-fold. The *erg7* gene encodes lanosterol synthase and experienced the biggest reduction. However, the *erg3* gene was upregulated by 11.64-fold. It was reported that the last reactions in ergosterol biosynthesis are catalyzed by *erg3*/*erg4*/*erg5*, and these enzymes catalyze the conversion of episterol into ergosterol [36]. Therefore, increased expression of *erg3* may be considered to be a compensation response to the downregulation of *erg4*/*erg5*.

#### 2.5.3. DEGs Involved in Conidia

Asexual sporulation is fundamental to the ecology and lifestyle of fungi. The ability to produce conidia is a key factor contributing to the fecundity, propagation, and fitness of *A. flavus*. The formation and maturation of conidia is primarily governed by the *brlA-abaA-wetA* regulatory cascade [37,38]. In the current study, the *brlA*, *abaA*, and *wetA* genes were significantly downregulated to different degrees, directly leading to lower levels of conidia formation. In addition, the transcription of the hydrophobin genes *rodA* and *rodB* was remarkably downregulated (Table 1). The formation of rodlets, physical resistance, and immunological inertia of the conidia is partly due to the presence of a hydrophobic layer that is composed of a protein from the hydrophobin family [39]. These identified DEGs indicate that benzenamine hinders the normal formation and physiological state of *A. flavus* conidia.

#### 2.5.4. DEGs Involved in Transcription Factors

Numerous transcription factor-encoding genes that are related to fungal development were differentially expressed in *A. flavus* after exposure to benzenamine. Most of the transcription factors, such as the secondary metabolism regulator *laeA*, C_2_H_2_ finger domain transcription factor *sebA*, and Ca^2+^ regulator and membrane fusion protein *fig1*, were downregulated to varying extents, whereas some transcription factors were upregulated, including the C_2_H_2_-like transcription factor *mtfA* and the developmental and secondary metabolism regulator *veA*. Among these, *laeA* and *veA* are the most important regulatory genes in *Aspergillus* spp. A complicated network of global regulators governs the development and secondary metabolism in *Aspergillus* spp. by including *laeA* and *veA* [19]. *LaeA* was first identified in *Aspergillus nidulans*, and it has been extensively studied. Numerous developmental genes are regulated by *laeA*, such as *knh1*, encoding a GPI-anchored protein involved in cell wall biosynthesis; *stuA*, encoding a cell pattern formation-associated protein that is related to conidiophore development; and, hydrophobic proteins encoded by *rodA/B* [40]. *VeA* also has the ability to regulate developmental genes, such as the downregulation of the asexual development-associated transcription factors *brlA* and *abaA*, which are required for the formation of conidia [38,41]. A heterotrimeric complex that is formed by the proteins encoded by *laeA*, *veA*, and *velB* has been reported to regulate sporulation and secondary metabolism [42]. In addition, it was reported that *laeA* and *veA* negatively affect each other’s transcription [19,43].

The transcriptomic results reveal that benzenamine exerted different effects on the expression of *laeA* and *veA* in the current work. The gene *laeA* was significantly downregulated by 3.57-fold, whereas *veA* was upregulated by 2.67-fold. This phenomenon may be due to the opposing regulatory effects of *laeA* and *veA*. The results indicate that benzenamine may suppress the development of *A. flavus* through its adverse effects on key processes, such as cell wall synthesis and conidia production, by regulating the expression of *laeA* and *veA*. This is also consistent with the above analysis results. In addition, the gene *cytC*, which encodes an apoptogenic factor, was significantly upregulated (5.03-fold). We hypothesize that *cytC* may be regulated by *laeA* or *veA*. However, up to now, regulation by *laeA* or *veA* of genes that are related to apoptosis has not been described, and we will investigate this inference in our future work.

### 2.6. Analysis of DEGs Involved in Aflatoxin Biosynthesis

#### 2.6.1. DEGs Involved in Aflatoxin Biosynthesis

To evaluate the regulatory roles of benzenamine on aflatoxin biosynthesis, the expression levels of genes that are involved in aflatoxin biosynthesis were analyzed. The biosynthetic pathways of aflatoxin have been well described [44,45]. A total of 10 genes that are involved in the aflatoxin biosynthesis, especially *aflA*, *aflB*, *aflD*, *aflT*, and *aflU*, were downregulated by benzenamine (Table 2). Two fatty acid synthases, *aflA* and *aflB*, are related to the early stage of aflatoxin biosynthesis, and they are capable of converting acetate to norsolorinic acid (NOR), which is a stable aflatoxin precursor [46,47]. On the other hand, there was no significant difference in the expression of *aflC*, which has an equivalent function. The expression level of *aflD* was reported to play a significant role in aflatoxin biosynthesis. The gene *aflD* encodes a norsolorinic acid ketoreductase that converts NOR to averantin (AVN) [48]. Additionally, *aflE* and *aflF*, homologous to *aflD*, are predicted to catalyze NOR to AVN [47,49]. The expression of *aflF* was downregulated by 3.13-fold, and *aflE* showed no significant difference in expression. *AflT* encodes a transmembrane protein and it is located at the end of the gene cluster for aflatoxin biosynthesis [50]. It was reported that *aflT* is not essential for the production and secretion of aflatoxin, and the expression of *aflT* is not regulated by the transcription regulator genes *aflR* and *aflS,* but by *fadA*, which encodes a G alpha protein-dependent signaling pathway [51]. Furthermore, *aflR* encodes a specific zinc-finger DNA-binding protein, which is an important regulatory gene that is required for transcriptional activation of most genes in aflatoxin biosynthesis [47,52]. The downregulation of *aflR* by benzenamine could cause changes in other aflatoxin biosynthesis pathway genes. However, the expression level of another regulatory gene, *aflS*, did not display any obvious changes. Our data clearly demonstrate that the aflatoxin production of *A. flavus* treated by benzenamine is directly reduced by downregulating the expression levels of aflatoxin biosynthesis genes.

#### 2.6.2. DEGs Involved in Carbon/Nitrogen Metabolism

It has been reported that aflatoxin production is influenced by nutrition factors, such as carbon and nitrogen sources [53,54]. Several DEGs that are involved in carbon and nitrogen metabolism were found in our data. Carbon catabolite repression (CCR) is a regulatory phenomenon that is hierarchically implemented to organize carbohydrate utilization, which is required for the regulation of growth and secondary metabolism in fungi [55,56]. *CreA*, which is a global regulator of CCR, encodes a zinc finger of a Cys2/His2 class protein and mediates various alternative carbon-utilizing systems [57,58]. The deletion of *creA* induces a strong reduction of aflatoxin synthesis. Additionally, cell wall homeostasis and conidial differentiation are regulated by *creA* [54,59]. The expression levels of the *creA* transcript in *A. flavus* after exposure to benzenamine were significantly lower than in the control. It is supposed that *creA* regulates gene expression by binding to consensus binding sites in the promoters of target genes, and this consensus binding site has been found in most aflatoxin gene promoter regions [60,61]. It appears likely that the downregulation of *creA* by benzenamine also contributes to the depression of aflatoxin production.

Nitrogen source is another important nutritional factor that is linked with aflatoxin biosynthesis [62,63]. Microorganisms can use a wide range of nitrogen sources, and different nitrogen sources may have different effects on aflatoxin production [53]. For example, it has been reported that glutamine and tyrosine favor aflatoxin production in *A. flavus*, while tryptophan does not [64,65]. Nitrogen utilization is often mediated by nitrogen metabolite repression (NMR) [66]. The gene *nmrA* negatively regulates several genes that are involved in NMR and it appears to be involved in the development and aflatoxin biosynthesis in *A. flavus*. Furthermore, the absence of *nmrA* results in reduced aflatoxin production [67]. The results of RNA-Seq in our study show that the expression of *nmrAL1*, which encodes *nmrA*-like family domain-containing protein 1, was significantly decreased. Additionally, *gad1* and *gfa1*, which are involved in the glutamine metabolic process, were downregulated by benzenamine.

#### 2.6.3. Other Related DEGs

Previous reports have demonstrated that pathway-specific regulators, as well as a complicated network of global regulators, govern multiple secondary metabolite gene clusters [19,43]. The expression of aflatoxin biosynthesis cluster genes is also modified by the global regulator *laeA*. The deletion of *laeA* blocks the production of aflatoxin by downregulating the expression of early aflatoxin biosynthesis genes and the pathway-specific transcriptional regulator *aflR* [18]. In addition, conidial development and aflatoxin formation are tightly coordinated in *A. flavus* [43]. The loss of conidial hydrophobicity in the *laeA* deletion mutant is considered to be capable of influencing the formation and stability of vesicles, thereby reducing aflatoxin biosynthesis [18,68]. Additionally, the loss of *laeA* may downregulate the expression of *nmrA*, and this regulation of *nmrA* contributes to reduced aflatoxin biosynthesis [67]. Our data agree with those indicating comprehensive regulation in *A. flavus* by *laeA*.

The cAMP/PKA signaling pathway regulates fungal morphogenesis and metabolism, including mycotoxin biosynthesis [69,70,71,72]. Previous papers have shown that cAMP signaling plays an important role in hyphal growth, conidiation, and production of DON [73,74]. It was reported that decreasing levels of cAMP block aflatoxin biosynthesis in *A. flavus* [75,76]. In the current work, all three DEGs in the cAMP signaling pathway were downregulated by magnitudes that ranged from 2.02- to 4.30-fold. The results indicate that the downregulation of the cAMP pathway genes by benzenamine is likely to negatively regulate aflatoxin biosynthesis in *A*. *flavus*. Additionally, of interest, most of the genes that are involved in purine metabolism were significantly downregulated by benzenamine, which implies that this process might have a role in aflatoxin synthesis.

The RNA-Seq data indicate that benzenamine not only directly reduces the production of aflatoxin by downregulating the aflatoxin biosynthesis pathway genes and pathway-specific regulatory genes, but it also indirectly blocks aflatoxin synthesis by mediating nutrient metabolism, signaling pathways, and the expression of the global transcription regulator.

### 2.7. Analysis of DEGs Involved in the Virulence of A. flavus

#### 2.7.1. DEGs Involved in Hydrolases

Extracellular hydrolases, such as glucosidase, proteases, and lipases, are critical for *A. flavus* to colonize its hosts [49,77,78]. *A. flavus* is able to degrade complex organic substrates, obtain nutrients for growth, macerate, and then invade host tissues [79]. The decreased abundance of hydrolases increases the difficulty for mycelia to penetrate and colonize hosts [40]. Several *A. flavus* hydrolytic enzymes, including α-glucosidase, lipase, and neutral protease, were downregulated to different degrees, according to our results (Table 3). Additionally, cutinase transcription factors *ctf1A/B* showed significantly downregulated transcription. However, of interest, there was no significant difference in the expression of cutinase in *A. flavus* after exposure to benzenamine.

#### 2.7.2. DEGs Involved in the Development and Metabolism of *A. flavus*

The virulence of *A. flavus* has been proved to be multifactorial, and it is also tightly coordinated with development, sporulation, and metabolism [80]. The cell wall is critical for the virulence of fungal pathogenicity [25]. Cell wall components, such as polysaccharides and proteins, are considered to be virulence factors and they contribute to colonization of the host [81,82]. The loss of cell wall integrity might influence the colonization by *A. flavus* of the host [75]. Several genes that are involved in the cell wall were downregulated in our study. Therefore, benzenamine might reduce the virulence of *A. flavus* by damaging its cell wall integrity.

*Aspergillus* species have the ability to produce a large quantity of asexual spores that spread through conidia [83]. Conidia are abundantly suspended in the air and environment, and they can remain viable for a long period of time [84,85]. These conidia will form a short germ tube and germinate when they colonize in hosts [86]. The formation and germination of conidia is critical for successful colonization. The expression of *stuA*, which encodes a cell pattern formation-associated protein that is involved in conidiophore development, was downregulated by benzenamine. Additionally, in *Aspergillus*, conidial hydrophobicity plays an important role in the infection of host tissues. The insoluble hydrophobic rodlet layer that is enveloped in the surface of *A. flavus* conidia contributes to the strengthening of the dispersal capacity and survival in a hostile environment, and the rodlet layer comprises the hydrophobic *rodA* protein that covalently binds to the conidial cell wall via GPI remnants [39,87]. Previous studies have reported that decreased conidial hydrophobicity accompanies reduced pathogenicity [88]. Our data demonstrate that hydrophobins *rodA* and *rodB* were also significantly decreased in abundance. The results indicate that conidial hydrophobins may be possible targets for preventing *A. flavus* infection in maize, which is in agreement with the above statement.

Carbon and nitrogen nutrients are required for the growth and secondary metabolism of fungi, and the effect of these nutrients on the virulence of *A. flavus* was reported recently. *CreA* and *nmrA* play important roles in the invasive virulence of *A. flavus*. The deletion of *creA* causes a defect in its capacity to effectively infect the host due to a reduction in conidial quantity and hydrophobicity [54]. Similarly, the loss of *nmrA* decreases the virulence of *A. flavus*, compromising its ability to produce conidia and colonize the host [67]. Thus, according to our data, benzenamine might reduce the pathogenicity of *A. flavus* by decreasing the expression of *creA* and *nmrA*.

In addition, the global regulator *laeA* and cAMP signaling have been reported to regulate the virulence of *A. flavus*. Previous studies reported that the deletion of *laeA* decreases the ability to colonize seeds [40,43]. In *laeA* mutant strains, the expression of several genes that are vital for pathogenicity (such as lipase, α-amylase, *nmrA*, and *rodA*) is downregulated, which might result in reduced fungal virulence [18,67]. The involvement of the cAMP signaling pathway in the regulation of fungal virulence has been reported [89,90]. The loss of genes in the cAMP signaling pathway, such as *acyA* and *cpk1*, considerably reduces the virulence of pathogenic fungi by repressing conidial production [74,76,91]. In the present study, our data demonstrate that benzenamine could decrease the virulence of pathogenic *A. flavus* by regulating *laeA* transcription and the cAMP signaling pathway.

### 2.8. Validation of RNA-Seq Data by qRT-PCR

**Figure 6:**
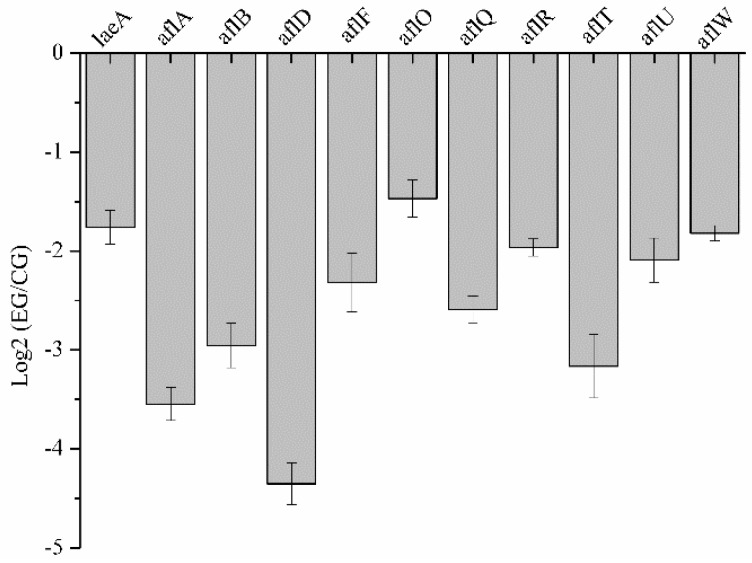
Quantitative real-time PCR validation of aflatoxin biosynthesis genes and *laeA* after treatment with benzenamine. CG, control group; EG, experimental group. Results are presented as mean ± SD. Log2 (EG/CG) ≤ −1 indicates downregulated expression.

**Figure 7:**
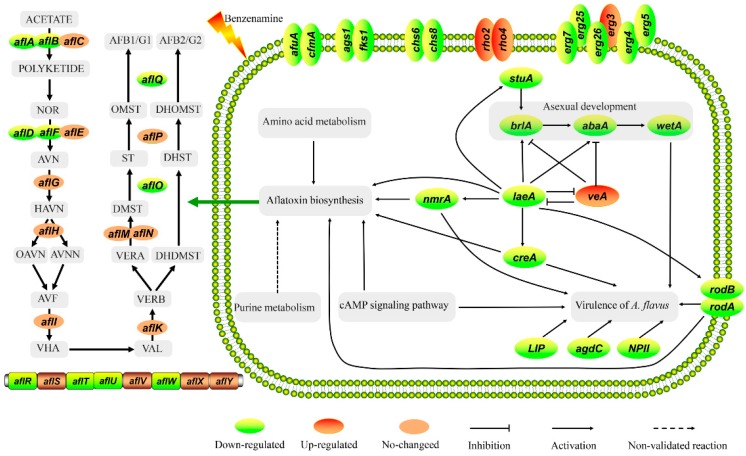
A schematic diagram for the proposed mechanism of benzenamine against *Aspergillus flavus*.

The qRT-PCR experiment was used to validate the RNA-Seq data in our study. DEGs that are involved in aflatoxin synthesis and the important global regulatory factor *laeA* were chosen for qRT-PCR validation. The differential gene expression profiles between the control group (CG) and experimental group (EG) are shown in Figure 6. The results show that these genes have expression patterns that are consistent with the RNA-Seq data, indicating the reliability of the transcriptome analysis in the current work. Overall, in order to elucidate the regulatory molecular events following *A. flavus* exposure to benzenamine, a hypothetical molecular mode of action is proposed on the basis of our data (Figure 7).

## 3. Conclusions

The current work demonstrates that benzenamine exerts strong inhibitory effects on the development, aflatoxin production, and pathogenicity of *A. flavus*, and the results provide fundamental information in understanding the fungal response to benzenamine at the transcriptional level. Based on the transcriptional profile, thousands of genes are downregulated in *A. flavus* after treatment with benzenamine, including genes that are associated with growth, differentiation, and aflatoxin biosynthesis in *A. flavus*, and we conclude that benzenamine inactivates *A. flavus* by suppressing the expression of related genes by downregulating the regulatory factor *laeA*. These enriched DEGs could be exploited in order to develop genetic strategies to reduce contamination by pathogenic *A. flavus*.

## 4. Materials and Methods

### 4.1. Microorganisms

*A. flavus* used in this study was obtained from the China Center of Industrial Culture Collection (CICC NO. 2219). The strain was cultured on PDA (200 g/L potato infusion, 20 g/L dextrose, and 20 g/L agar) in Petri Dishes at 28 °C for three days. Conidia were washed with sterile distilled water containing 0.05% (*v*/*v*) Tween 80, and their density was adjusted by a hemocytometer to a final concentration of approximately 1 × 10^6^ conidia/mL.

### 4.2. Antifungal Assays

#### 4.2.1. Inhibition of Hyphal Growth of *A. flavus*

To study the efficacy of benzenamine against mycelial growth, a setup of two inverse face-to-face Petri Dishes was applied according to Wu et al. [14]. The system consists of two 6 cm lidless Petri Dishes. The upper plate contained 5 mL of PDA inoculated with a 6 mm diameter *A. flavus* agar plug in the center, and the lower plate contained benzenamine at different concentrations (25, 50, 100, 200, 400 µL/L). The two dishes were sealed with a double layer of parafilm and then incubated at 28 °C for three days. The treatments consisted of three replicates, and each experiment was performed in triplicate. The inhibition rate of mycelial growth was calculated using the following Equation:

Inhibition rate (%) = (Rc − Rt)/Rc × 100%(1)

where Rc is the colony diameter of the control and Rt is the colony diameter of the treatment.

#### 4.2.2. Inhibition of Conidial Germination of *A. flavus*

In this assay, face-to-face Petri Dishes, as described above, were applied. The top dishes, which contained a sterile filter-paper dipped in benzenamine, were attached face-to-face to a PDA plate spread with a 100 μL conidial suspension of *A. flavus*. The setup was then incubated at 28 °C for 9 h. Conidial germination was evaluated by examining no less than 100 conidia per Petri Dish. Conidia were considered to be germinated when the germ tube measured at least twice its length [92]. The treatments consisted of three replicates, and each experiment was performed in triplicate. Inhibition of germination was calculated according to the formula:

Inhibition of germination (%) = (Gc − Gt)/Gc × 100%(2)

where Gc is the germination rate of the control and Gt is the germination rate of the fungus exposed to benzenamine.

### 4.3. Determination of Aflatoxin B1

Aflatoxin B1 content was detected with an ELISA kit in this study. *A. flavus* was treated with 100 µL/L of benzenamine for five days at 28 °C. Aflatoxin B1 extraction was carried out following the procedure that was described by Bavaro et al. [93]. Briefly, five fungal agar plugs (6 mm diameter) were extracted with 25 mL of 80% methanol. The extraction solution was centrifuged at 4500 rpm for 10 min and the supernatant was measured using an ELISA kit (Huaan Magnech Bio-Tech Co., Ltd., Beijing, China), according to the manufacturer’s instructions. The linear range was between 0.03–2 ng/g for AFB1 (*R*^2^ = 0.9983). The limit of detection (LOD) was 0.03 ng/g and the limit of quantification (LOQ) was 0.08 ng/g. The average spiked recovery rate was 100 ± 20%, with the coefficient of variation (CV) being less than 10%.

### 4.4. Virulence of A. flavus in Maize

Undamaged maize kernels were surface sanitized with 0.1% hypochlorite and then rinsed with sterile water three times. The maize kernels inoculated with the conidial suspension (1 × 10^6^ conidia/mL) of *A. flavus* were placed in a lidless plate, and then 100 μL/L of benzenamine was distributed evenly on the inner surface of the plate in the form of small droplets (0.5 μL). The two dishes were sealed and incubated at 28 °C for five days, after which the fungal colonization of maize kernels was observed on each plate and the colonized kernels were harvested in 100 mL conical flasks with 20 mL of sterile 0.05% Tween 80 water solution. Spores were counted by a hemocytometer.

### 4.5. Preparation of cDNA Libraries and Illumina Sequencing

The conidial suspension of *A. flavus* was spread on PDA medium that was overlaid with sterile cellophane and then sequentially exposed to 100 µL/L of benzenamine for five days at 28 °C. The cellophane membrane with mycelia from the whole plate was scraped off with a knife and then immediately frozen in liquid nitrogen for RNA extraction. Total RNA was isolated using Trizol Reagent (Invitrogen Life Technologies, Carlsbad, CA, USA), according to the manufacturer’s instructions. The concentration and quality of RNA for each sample were determined by a NanoDrop 2000 spectrophotometer (Thermo Scientific, Wilmington, DE, USA), and the integrity of RNA was checked using an Agilent 2100 Bioanalyzer (Agilent Technologies, Palo Alto, CA, USA).

The mRNA was enriched from total RNA using poly-T oligo-attached magnetic beads and then cleaved into short fragments using divalent cations under an elevated temperature in an Illumina proprietary fragmentation buffer. The cleaved mRNA fragments were copied into first-strand cDNA using random oligonucleotides and Super Script II, followed by second-strand cDNA synthesis using DNA Polymerase I and RNase H. The remaining overhangs were converted into blunt ends via exonuclease/polymerase activities, and the enzymes were then removed. After adenylation of the 3′ ends of the DNA fragments, Illumina PE adapter oligonucleotides were ligated to prepare for hybridization. The adaptor-modified fragments were purified with the AMPure XP system (Beckman Coulter, Beverly, CA, USA) in order to preferentially select 200 bp in length. DNA fragments with ligated adaptor molecules on both ends were enriched using the Illumina PCR Primer Cocktail in a 15-cycle PCR reaction. The products were purified with the AMPure XP system and then quantified using the Agilent high-sensitivity DNA assay on a Bioanalyzer 2100 system (Agilent Technologies, Palo Alto, CA, USA). The sequencing library was then sequenced with the Hiseq 2500 platform (Illumina, San Diego, CA, USA) by Shanghai Personal Biotechnology Cp. Ltd.

### 4.6. De Novo Transcriptome Assembly and Annotation

De novo transcriptome analysis was performed according to Diao et al. [94]. Briefly, the quality control analysis on raw data was done by using FastQC (Version 0.11.7, Babraham bioinformatics, Cambridge, UK, 2018). Subsequently, the clean reads were obtained by removing adaptors and low-quality reads with Cutadapt [95]. The clean reads were then assembled de novo using the Trinity platform (http://trinityrnaseq.sourceforge.net/). The longest transcripts of each gene were regarded as unigenes. All of the unigenes were assigned to five databases using BLASTx (*E*-value ≤ 10^−5^), including NR (NCBI non-redundant protein sequences), GO (Gene Ontology), KEGG (Kyoto Encyclopedia of Genes and Genome), eggNOG (evolutionary genealogy of genes: Non-supervised Orthologous Groups), and Swiss-Prot.

### 4.7. Identification and Analysis of DEGs

The levels of gene expression were estimated as normalized FPKM (fragments per kilobase of transcript per million mapped reads) using RSEM (RNA-seq by expectation-maximization) [96]. Differential expression analysis was performed with DESeq (Version 1.20.0, Bioconductor, New York, NY, USA, 2018) and genes with a fold change >2 and corrected *p*-value < 0.05 were set as the threshold for significantly differential expression. Finally, GO functional annotation and KEGG pathway enrichment analysis were performed to uncover the functions of DEGs.

### 4.8. qRT-PCR Analysis

To validate the reliability of *A. flavus* gene expression data obtained by RNA-Seq, qRT-PCR was conducted for 10 genes that were involved in aflatoxin biosynthesis and the global regulatory gene *laeA*. The primers are listed in Table S5; the *β-tubulin* gene was selected as the endogenous reference gene. Total RNA extraction was performed as described above, and cDNA was synthesized with a Takara RNA PCR Kit (Takara, Dalian, China). qRT-PCR was performed using an Mx3000p instrument (Stratagene, La Jolla, CA, USA) with a final volume of 20 μL containing 10 μL of SYBR premix ExTaq, 0.5 μL of each forward and reverse primer (10 mM), 2 μL of cDNA template, 0.4 μL of ROX Reference Dye, and 6.6 μL of RNase-free water. The comparative 2^−ΔΔCT^ method was employed to calculate relative gene expression [97].

### 4.9. Availability of Supporting Data

The raw data that was generated in this study has been deposited in the NCBI’s Sequence Read Archive (SRA) database with the accession number SRP181717 (BioProject ID: PRJNA516725).

### 4.10. Statistical Analyses

Statistical analyses were performed with SPSS for Windows version 20.0 (SPSS Inc., Chicago, IL, USA, 2011). The data were evaluated by Student’s t-test or one-way ANOVA followed by LSD according to the experimental design. *p* < 0.05 was considered to be statistically significant.

## Figures and Tables

**Table 1:** Transcript abundance of genes involved in *Aspergillus flavus* development.

Gene ID	Gene Name	Log2 (EG/CG) *	*p*-Value	Function
Cell wall				
TRINITY_DN6715_c0_g1	crh11	−3.98	8.95 × 10^−8^	cell wall glucanase
TRINITY_DN10780_c1_g3	ags1	−2.24	1.53 × 10^−5^	Alpha-1,3-glucan synthase
TRINITY_DN17727_c0_g1	fks1	−1.38	8.42 × 10^−3^	beta-1,3 -glucan synthase
TRINITY_DN9747_c0_g1	rho2	1.42	2.71 × 10^−4^	GTP-binding protein
TRINITY_DN9087_c0_g1	rho4	1.31	5.24 × 10^−3^	GTP-binding protein
TRINITY_DN11443_c2_g2	cfmA	−1.51	3.20 × 10^−4^	GPI-anchored CFEM domain protein A
TRINITY_DN11909_c4_g1	afuA	−1.75	2.47 × 10^−3^	GPI-anchored membrane protein
TRINITY_DN9375_c0_g1	chs6	−1.02	1.65 × 10^−3^	Chitin synthase
TRINITY_DN10195_c0_g1	chs8	−1.31	1.13 × 10^−3^	Chitin synthase
Cell membrane				
TRINITY_DN8232_c0_g1	erg3	3.58	1.91 × 10^−2^	C-5 sterol desaturase
TRINITY_DN10457_c0_g1	erg4	−2.87	8.85 × 10^−6^	C6 transcription factor
TRINITY_DN7343_c0_g1	erg5	−1.80	5.19 × 10^−3^	Cytochrome P450
TRINITY_DN6009_c0_g1	erg6	−1.23	4.12 × 10^−2^	24-C-methyltransferase
TRINITY_DN9690_c0_g1	erg7	−6.36	6.64 × 10^−21^	Lanosterol synthase
TRINITY_DN11958_c0_g2	erg13	−1.34	2.89 × 10^−2^	Hydroxymethylglutaryl-CoA synthase
TRINITY_DN10380_c0_g1	erg25	−1.64	1.37 × 10^−2^	Methylsterol monooxygenase
TRINITY_DN15242_c0_g1	erg26	−1.59	1.48 × 10^−3^	Sterol-4-alpha-carboxylate 3-dehydrogenase
Conidia				
TRINITY_DN11281_c0_g1	brlA	−1.27	8.69 × 10^−4^	C_2_H_2_ type master regulator of conidiophore development
TRINITY_DN11770_c0_g1	wetA	−3.99	1.85 × 10^−21^	developmental regulatory protein
TRINITY_DN9659_c0_g1	abaA	−3.17	1.23 × 10^−12^	Conidiophore development regulator
TRINITY_DN11518_c0_g4	rodA	−9.10	6.30 × 10^−19^	Conidial hydrophobin
TRINITY_DN15172_c0_g1	rodB	−6.94	4.97 × 10^−23^	Conidial hydrophobin
TRINITY_DN11879_c4_g1	stuA	−1.54	7.43 × 10^−6^	Cell pattern formation-associated protein
Transcription regulator				
TRINITY_DN9345_c0_g1	laeA	−1.82	5.63 × 10^−5^	Secondary metabolism regulator
TRINITY_DN11910_c4_g1	veA	1.42	2.68 × 10^−3^	Developmental and secondary metabolism regulator
TRINITY_DN8021_c0_g1	fig1	−2.59	4.64 × 10^−3^	Ca^2+^ regulator and membrane fusion protein
TRINITY_DN10388_c0_g1	sebA	−2.44	2.36 × 10^−4^	C_2_H_2_ finger domain transcription factor
TRINITY_DN12068_c7_g5	mtfA	1.06	2.65 × 10^−2^	C_2_H_2_ finger domain transcription factor
TRINITY_DN12136_c0_g2	hir3	−1.57	1.24 × 10^−3^	Histone transcription regulator
Developmental signal				
TRINITY_DN7900_c0_g1	fluG	−2.25	3.28 × 10^−5^	Extracellular developmental signal biosynthesis protein
Apoptosis				
TRINITY_DN12180_c5_g1	cycA	2.33	5.30 × 10^−5^	Cytochrome c
G-protein				
TRINITY_DN10123_c0_g1	gblP	−1.90	1.37 × 10^−6^	Nucleotide-binding protein subunit beta-like protein
TRINITY_DN9739_c0_g1	gna12	−2.16	2.48 × 10^−6^	G-protein alpha subunit

(*): CG, control group; EG, experimental group. Log2 (EG/CG) ≥1 indicate up-regulated expression and Log2 (EG/CG) ≤−1 indicate down-regulated expression.

**Table 2:** Transcript abundance of genes that are involved in aflatoxin biosynthesis.

Gene ID	Gene Name	Log2 (EG/CG) *	*p*-Value	Function
Aflatoxin biosynthesis				
TRINITY_DN9945_c0_g1	aflA	−3.42	5.80 × 10^−10^	Fatty acid synthase alpha subunit
TRINITY_DN6952_c0_g1	aflB	−3.32	4.61 × 10^−2^	Fatty acid synthase subunit beta
TRINITY_DN61_c0_g1	aflD	−4.96	2.64 × 10^−8^	Norsolorinic acid ketoreductase
TRINITY_DN12173_c2_g2	aflF	−1.65	4.36 × 10^−2^	Norsolorinic acid ketoreductase
TRINITY_DN10698_c0_g2	aflO	−1.83	5.63 × 10^−3^	*O*-methyltransferase
TRINITY_DN10938_c0_g1	aflQ	−1.49	2.16 × 10^−4^	Oxidoreductase
TRINITY_DN10167_c0_g1	aflR	−1.86	1.68 × 10^−2^	Aflatoxin biosynthesis regulatory protein
TRINITY_DN9418_c0_g1	aflT	−2.72	4.27 × 10^−2^	Transmembrane protein
TRINITY_DN399_c0_g1	aflU	−3.24	2.31 × 10^−8^	P450 monooxygenase
TRINITY_DN10373_c0_g1	aflW	−1.60	1.37 × 10^−2^	FAD-binding monooxygenase
Carbon metabolism				
TRINITY_DN7925_c1_g1	creA	−1.14	9.62 × 10^−5^	DNA-binding protein
TRINITY_DN10031_c0_g1	mexAM	1.55	1.84 × 10^−2^	Oxidoreductase
TRINITY_DN10394_c0_g1	pot1	1.90	1.45 × 10^−6^	Acetyl-CoA C-acyltransferase
TRINITY_DN11110_c0_g1	rntA	1.88	5.21 × 10^−6^	Guanyl-specific ribonuclease
TRINITY_DN11497_c0_g1	ppoA	−1.83	7.67 × 10^−6^	Linoleate 8R-dioxygenase like protein
TRINITY_DN12116_c3_g3	ppoC	−2.79	9.25 × 10^−4^	Fatty acid oxygenase
Nitrogen metabolism				
TRINITY_DN5952_c0_g1	nmrAL1	−1.60	2.14 × 10^−4^	NmrA-like family domain-containing protein 1
TRINITY_DN11972_c8_g2	gdh-1	−2.42	1.52 × 10^−9^	Specific glutamate dehydrogenase
TRINITY_DN11667_c1_g3	glt1	−2.40	4.57 × 10^−5^	Putative glutamate synthase
TRINITY_DN11707_c0_g1	niiA	−1.73	4.23 × 10^−8^	Nitrite reductase
TRINITY_DN10046_c0_g1	nirA	−1.93	2.28 × 10^−4^	Nitrogen assimilation transcription factor
TRINITY_DN13321_c0_g1	ddc	1.36	1.02 × 10^−2^	Aromatic-L-amino-acid decarboxylase
TRINITY_DN9125_c0_g1	aat2	−3.66	1.66 × 10^−2^	Aspartate aminotransferase
TRINITY_DN19445_c0_g1	melO	−3.40	8.11 × 10^−8^	Tyrosinase
TRINITY_DN10664_c0_g1	orsC	−1.06	4.78 × 10^−2^	Tyrosinase-like protein
TRINITY_DN11859_c0_g1	gad1	−4.38	1.27 × 10^−27^	Glutamate decarboxylase
TRINITY_DN11916_c1_g2	gfa1	−1.46	2.29 × 10^−4^	Glutamine--fructose-6-phosphate aminotransferase
TRINITY_DN12169_c4_g1	sch9	1.86	2.72 × 10^−4^	Serine/threonine-protein kinase
Purine metabolism				
TRINITY_DN12187_c1_g2	ade17	−1.58	6.35 × 10^−5^	Bifunctional purine biosynthesis protein
TRINITY_DN8936_c0_g1	uaY	−3.71	2.76 × 10^−4^	Positive regulator of purine utilization
TRINITY_DN9209_c0_g1	uapC	−3.11	1.58 × 10^−9^	Purine permease
TRINITY_DN9614_c0_g1	fcy2	−2.36	3.47 × 10^−4^	Purine-cytosine permease
TRINITY_DN10376_c0_g2	pol12	−2.28	3.34 × 10^−2^	DNA polymerase alpha subunit B
TRINITY_DN8807_c0_g1	hxA	−1.16	2.02 × 10^−3^	Xanthine dehydrogenase
cAMP signaling pathway				
TRINITY_DN10852_c0_g2	ATP12A	−2.10	8.03 × 10^−3^	ATPase alpha 1 subunit
TRINITY_DN11913_c3_g2	pld1	−1.55	8.45 × 10^−5^	Phospholipase D1
TRINITY_DN11065_c0_g2	pka-C3	−1.02	1.11 × 10^−2^	cAMP-dependent protein kinase

(*): CG, control group; EG, experimental group. Log2 (EG/CG) ≥ 1 indicate up-regulated expression and Log2 (EG/CG) ≤ −1 indicate down-regulated expression.

**Table 3:** Transcript abundance of genes that are involved in virulence of *Aspergillus flavus*.

Gene ID	Gene Name	Log2 (EG/CG) *	*p*-Value	Function
Hydrolase				
TRINITY_DN11687_c2_g1	mal1	−3.39	6.39 × 10^−10^	Alpha-glucosidase
TRINITY_DN18434_c0_g1	mal2	−2.86	2.02 × 10^−3^	Alpha-glucosidase
TRINITY_DN9284_c0_g1	agdC	−2.16	5.88 × 10^−6^	Alpha -glucosidase
TRINITY_DN4002_c0_g1	lip	−3.41	2.15 × 10^−3^	Lipase
TRINITY_DN11096_c0_g3	NPII	−7.81	2.84 × 10^−61^	Neutral protease
TRINITY_DN10289_c0_g1	ctf1B	−2.77	2.89 × 10^−2^	Cutinase transcription factor 1 beta
TRINITY_DN11791_c0_g4	ctf1A	−1.59	4.29 × 10^−2^	Cutinase transcription factor 1 alpha
TRINITY_DN8062_c0_g1	abfA	−2.71	2.69 × 10^−3^	Alpha-L-arabinofuranosidase A
TRINITY_DN12177_c2_g1	dvrA	1.03	1.97 × 10^−2^	C_2_H_2_ finger domain transcription factor

(*): CG, control group; EG, experimental group. Log2 (EG/CG) ≥1 indicate up-regulated expression and Log2 (EG/CG) ≤−1 indicate down-regulated expression.

## Supplementary Materials

The following are available online at http://www.mdpi.com/2072-6651/11/2/70/s1, Table S1: Statistics on filtering of RNA-Seq Data, Table S2: Summary of transcripts and unigenes in this study, Table S3: Summary of annotation results, Table S4: Summary of differentially expressed genes, Table S5: List of primers used in this study.
