# Supplementary material for: Transcriptomic Insights into Benzenamine Effects on the Development, Aflatoxin Biosynthesis, and Virulence of Aspergillus flavus
Source: Toxins (Basel). 2019 Jan 27;11(2):70. doi: 10.3390/toxins11020070 (PMC6410012; doi:10.3390/toxins11020070)
Supplement: Supplementary file 1 [file toxins-11-00070-s001.zip › toxins-426585-SI/final-supplementary (toxins-426585).docx]

Supplementary Materials: Transcriptomic Insights into Benzenamine Effects on the Development, Aflatoxin Biosynthesis, and Virulence of *Aspergillus flavus*

Mingguan Yang, Laifeng Lu, Shuhua Li, Jing Zhang, Zhenjing Li, Shufen Wu, Qingbin Guo, Huanhuan Liu and Changlu Wang

**Table S1.** Statistics on filtering of RNA-Seq Data.

| **Sample** | **CG1** | **CG2** | **CG3** | **EG1** | **EG2** | **EG3** |
| --- | --- | --- | --- | --- | --- | --- |
| Clean Reads NO. (million) | 44.71 | 42.86 | 42.39 | 44.44 | 40.82 | 44.69 |
| Clean Data (million bp) | 6740.74 | 6459.38 | 6391.06 | 6697.79 | 6154.46 | 6737.38 |
| Clean Reads % | 99.39 | 99.09 | 99.41 | 99.47 | 99.42 | 99.21 |
| Clean Data % | 99.24 | 98.91 | 99.26 | 99.27 | 99.27 | 99.04 |

CG, EG denoted the *Aspergillus flavus* in the absence and presence of benzenamine, respectively.

**Table S2.** Summary of transcripts and unigenes in this study.

| **Parameter** | **Contig** | **Transcript** | **Unigene** |
| --- | --- | --- | --- |
| Total Length (million bp) | 38.92 | 196.61 | 33.97 |
| Sequence Number | 59,359 | 63,660 | 23,639 |
| Max. Length (bp) | 27,446 | 29,290 | 29,290 |
| Mean Length (bp) | 656 | 3088 | 1437 |
| N50 (bp) | 2,773 | 2773 | 2969 |
| N50 Sequence No. | 3654 | 12,335 | 3336 |
| N90 (bp) | 162 | 1781 | 523 |
| N90 Sequence No. | 29,635 | 36,292 | 13,087 |
| GC % | 49.14 | 49.35 | 49.43 |

**Table S3.** Summary of annotation results.

| **Database** | **Number** | **Percentage** |
| --- | --- | --- |
| NR | 14,684 | 62.12 |
| GO | 9,940 | 42.05 |
| KEGG | 2,952 | 12.49 |
| eggNOG | 13,437 | 56.84 |
| Swissprot | 11,435 | 48.37 |
| In all database | 2,722 | 11.51 |

**Table S5.** List of primers used in this study.

| **Gene** | **Primer Sequence (5'–3')** | **PCR Product (bp)** |
| --- | --- | --- |
| β-tubulin | Forward primer 5′-ATGGCTGCTTCTGACTTCCG-3′ | 159 |
|  | Reverse primer 5′-CGCATCTGGTCCTCAACCTC-3′ |  |
| laeA | Forward primer 5′-TTATTCACGGTGGCAAGGG-3′ | 139 |
|  | Reverse primer 5′-CAACAACGAAAGCGTCTGG-3′ |  |
| aflA | Forward primer 5′-AGATTCGTCCGCTGTCCC-3′ | 169 |
|  | Reverse primer 5′-ATCAGCCAGGCAAGTTCG-3′ |  |
| aflB | Forward primer 5′-TCTGATGGTCTCCGCTCTG-3′ | 192 |
|  | Reverse primer 5′-TGTCCCTGACGCTGAATGT-3′ |  |
| aflD | Forward primer 5′-GCTCCCGTCCTACTGTTTCA-3′ | 115 |
|  | Reverse primer 5′-CATAGTCGTGCATGTTGGTG-3′ |  |
| aflF | Forward primer 5′-CAATGTCATCGCGGTTCTT-3′ | 137 |
|  | Reverse primer 5′-GACGGCTTTCGCTATCCTA-3′ |  |
| aflO | Forward primer 5′-TCGGATTGGGATGTGGTC-3′ | 121 |
|  | Reverse primer 5′-CTAGAGTTATCGGCGTGTCG-3′ |  |
| aflQ | Forward primer 5′-ATGGTGTTCAAGCCAGAGCG-3′ | 172 |
|  | Reverse primer 5′-TGGGCGAGATGAAGAAGCAG-3′ |  |
| aflR | Forward primer 5′-AGTGCGAGGCAACGAAAAGG-3′ | 169 |
|  | Reverse primer 5′-GCGAGTCTGGGAGGAACGGA-3′ |  |
| aflT | Forward primer 5′-ATCATCTGCGTTCTCCTGG-3′ | 146 |
|  | Reverse primer 5′-ACGGTGGCGTTATCCTTC-3′ |  |
| aflU | Forward primer 5′-TCCGACCTCACGACGCATTA-3′ | 137 |
|  | Reverse primer 5′-TGGTAGACGCCCGAAGACGA-3′ |  |
| aflW | Forward primer 5′-CGGTTCTTCGCTGCACTA-3′ | 195 |
|  | Reverse primer 5′-ATTGATTCCCACCATTTCG-3′ |  |
